# Supplementary material for: Stable Isotope Analyses Reveal Impact of Fe and Zn on Cd Uptake and Translocation by Theobroma cacao
Source: Plants (Basel). 2024 Feb 17;13(4):551. doi: 10.3390/plants13040551 (PMC10893372; doi:10.3390/plants13040551)
Supplement: Supplementary file 1 [file plants-13-00551-s001.zip › plants-2801716-supplementary.pdf]

## Supplementary Information

To:

# Stable isotope analyses reveal impact of Fe and Zn on Cd uptake and translocation by *Theobroma cacao*

Rebekah E. T. Moore[a], Ihsan Ullah[b], Jim M. Dunwell[b], Mark Rehkämper[a]

[a]Department of Earth Science and Engineering, Imperial College London, London SW7 2BP, UK

[b]School of Agriculture, Policy and Development, University of Reading, Reading RG6 6EU, UK

**Table S1:** Composition of the half-strength Hoagland solutions with additional $CdCl_2$ for the eight treatments

| Component | Species | Concentration mg $L^{-1}$ | Concentration µmol $L^{-1}$ | Treatment # |
|---|---|---|---|---|
| **Major nutrients** | | | | |
| $KH_2PO_4$ | $K^+$ | 19.55 | 500 | All |
| | $PO_4^{3-}$ | 47.49 | 500 | All |
| | $H^+$ | 2.002 | 1000 | All |
| $KNO_3$ | $K^+$ | 97.75 | 2500 | All |
| | $NO_3^-$ | 155.0 | 2500 | All |
| $Ca(NO_3)_2{\cdot}4H_2O$ | $Ca_2^+$ | 100.2 | 2500 | All |
| | $NO_3^-$ | 310.0 | 5000 | All |
| $MgSO_4{\cdot}7H_2O$ | $Mg^{2+}$ | 24.31 | 1000 | All |
| | $SO_4^{2-}$ | 96.07 | 1000 | All |
| **Micronutrients** | | | | |
| $H_3BO_3$ | $H_3BO_3$ | 1.430 | 23.1 | All |
| Fe-EDTA[a,b] | $Fe^{3+}$ | 2.383 | 42.7 | 1, 2, 3, 6, 7 |
| | $EDTA^{4-}$ | 12.30 | 42.7 | 1, 2, 3, 6, 7 |
| $MnCl_2{\cdot}4H_2O$ | $Mn^{2+}$ | 0.253 | 4.6 | All |
| | $Cl^-$ | 0.326 | 9.2 | All |
| $ZnSO_4{\cdot}7H_2O$[a] | $Zn^{2+}$ | 0.025 | 0.38 | 1, 2, 3, 4, 5 |
| | $SO_4^{2-}$ | 0.037 | 0.38 | 1, 2, 3, 4, 5 |
| $CuSO_4{\cdot}5H_2O$ | $Cu^{2+}$ | 0.010 | 0.2 | All |
| | $SO_4^{2-}$ | 0.015 | 0.2 | All |
| $Na_2MoO_4{\cdot}2H_2O$ | $Na^+$ | 0.002 | 0.1 | All |
| | $MoO_4^{2-}$ | 0.003 | 0.04 | All |
| $CdCl_2$[c] | $Cd^{2+}$ | 2.248 | 20.0 | All |
| | $Cl^-$ | 2.836 | 40.0 | All |

[a]$C_{10}H_{12}FeN_2O_8$ [b]After an initial 28 days in the half-strength Hoagland solution without $CdCl_2$, the cacao seedlings were grown in eight different solutions for 14 days. These solutions included or excluded Fe-EDTA and $ZnSO_4$. [c]The $CdCl_2$ was present in all eight final solutions but excluded from the solutions used for the initial 28 days.

**Table S2:** Cadmium concentrations and isotope compositions (relative to NIST SRM 3108 Cd) obtained for reference and quality control materials and comparison with literature data

| **Material** | **Type** | **n** | **[Cd] ± sd**<br>μg g$^{-1}$ | **$\delta^{114/110}$Cd ± 2sd**<br>‰ | **Reference** |
|---|---|---|---|---|---|
| BAM-I012 | Cd Solution | 4 | **-** | **–1.32±0.06** | **This study** |
| | | | - | –1.33±0.04 | [1] |
| | | | - | –1.32±0.06 | [2] |
| | | | - | –1.33±0.06 | [3] |
| NIST SRM 1570a | Spinach Leaf | 2 | **2.76±0.02** | **0.47±0.03** | **This study** |
| | | | 2.87±0.06 | - | Certificate |
| | | | 2.71±0.01 | 0.44±0.06 | [2] |
| | | | - | 0.47±0.03 | [3] |
| | | | 2.87±0.02 | 0.45±0.05 | [4] |
| BCR-679 | White Cabbage | 1 | **1.69** | **0.22±0.04** | **This study** |
| | | | 1.66±0.07 | - | Certificate |
| | | | 1.77±0.05 | 0.22±0.02 | [5] |

**Table S3:** Percentages of species present in the hydroponic solutions with or without Fe-EDTA and $ZnSO_4$, calculated using Visual MINTEQ 3.1

| Species[a] | Fe-EDTA + $ZnSO_4$ | Fe-EDTA | $ZnSO_4$ | Neither |
|---|---|---|---|---|
| $Cd^{2+}$ | 87.7 | 87.1 | 88.3 | 88.3 |
| $CdCl^{+}$ | 0.46 | 0.46 | 0.46 | 0.46 |
| $CdSO_4$ (aq) | 6.20 | 6.16 | 6.25 | 6.24 |
| $Cd(SO_4)_2^{2-}$ | 0.07 | 0.06 | 0.07 | 0.07 |
| $CdNO_3^{+}$ | 1.24 | 1.24 | 1.25 | 1.25 |
| $CdHPO_4$ (aq) | 3.62 | 3.59 | 3.64 | 3.64 |
| $CdEDTA^{2-}$ | 0.66 | 1.38 | | |
| $Cu^{2+}$ | 32.5 | 19.4 | 83.6 | 83.6 |
| $CuOH^{+}$ | 0.12 | 0.07 | 0.31 | 0.31 |
| $CuSO_4$ (aq) | 2.24 | 1.34 | 5.78 | 5.78 |
| $CuNO_3^{+}$ | 0.47 | 0.28 | 1.21 | 1.21 |
| $CuHPO_4$ (aq) | 3.52 | 2.10 | 9.17 | 9.07 |
| $CuEDTA^{2-}$ | 60.3 | 75.7 | | |
| $CuHEDTA^{-}$ | 0.89 | 1.12 | | |
| $Fe\text{-}EDTA^{-}$ | 99.1 | 99.4 | | |
| FeHEDTA (aq) | 0.02 | 0.02 | | |
| $FeOHEDTA^{2-}$ | 0.35 | 0.35 | | |
| $Fe(OH)_2^{+}$ | 0.17 | 0.08 | | |
| $FeHPO_4^{+}$ | 0.36 | 0.17 | | |
| $Zn^{2+}$ | 90.8 | | 91.3 | |
| $ZnSO_4$ (aq) | 5.97 | | 6.01 | |
| $Zn(SO_4)_2^{2-}$ | 0.04 | | 0.04 | |
| $ZnNO_3^{+}$ | 1.05 | | 1.05 | |
| $ZnHPO_4$ (aq) | 1.53 | | 1.53 | |
| $ZnEDTA^{2-}$ | 0.56 | | | |
| $ZnCl^{+}$ | 0.01 | | 0.02 | |
| $ZnOH^{+}$ | 0.01 | | | |

[a] The speciation of other elements was not significantly changed by the addition or removal of Fe-EDTA or $ZnSO_4$ and they are therefore omitted. Fe-EDTA and $ZnSO_4$ were both present in treatments 1, 2 and 3. Fe-EDTA, but not $ZnSO_4$, was present in treatments 6 and 7. $ZnSO_4$, but not Fe-EDTA, was present in treatments 4 and 5. Neither were present in treatment 8.

**Table S4:** Dry masses of plant tissue samples

| T # | Nutrient Solutions Hydroponic | Foliar | Roots g | Stems g | Leaves g | Total g | Average Total g | sd g |
|---|---|---|---|---|---|---|---|---|
| C | Fe, Zn | - | 0.10 | 0.17 | 0.38 | 0.65 | 0.69 | 0.27 |
| | | | 0.09 | 0.20 | 0.16 | 0.45 | | |
| | | | 0.09 | 0.17 | 0.72 | 0.99 | | |
| 1 | Fe, Zn | - | 0.11 | 0.16 | 0.36 | 0.63 | 0.78 | 0.21 |
| | | | 0.13 | 0.20 | 0.59 | 0.92 | | |
| 2 | Fe, Zn | Fe | 0.15 | 0.17 | 0.52 | 0.84 | 0.91 | 0.11 |
| | | | 0.11 | 0.19 | 0.70 | 0.99 | | |
| 3 | Fe, Zn | Zn | 0.10 | 0.17 | 0.69 | 0.96 | 0.86 | 0.14 |
| | | | 0.12 | 0.12 | 0.52 | 0.76 | | |
| 4 | Zn | - | 0.09 | 0.11 | 0.36 | 0.56 | 0.67 | 0.15 |
| | | | 0.09 | 0.14 | 0.54 | 0.77 | | |
| 5 | Zn | Fe | 0.11 | 0.15 | 0.52 | 0.79 | 0.69 | 0.14 |
| | | | 0.10 | 0.14 | 0.36 | 0.59 | | |
| 6 | Fe | - | 0.08* | 0.18 | 0.57 | 0.84 | 0.74 | 0.13 |
| | | | 0.11 | 0.18 | 0.51 | 0.79 | | |
| | | | 0.10 | 0.13 | 0.36 | 0.59 | | |
| 7 | Fe | Zn | 0.11 | 0.16 | 0.52 | 0.79 | 0.76 | 0.04 |
| | | | 0.10 | 0.13 | 0.50 | 0.73 | | |
| 8 | - | - | 0.08 | 0.13 | 0.52 | 0.73 | 0.72 | 0.01 |
| | | | 0.09 | 0.19 | 0.44 | 0.72 | | |
| | | **Mean** | **0.11** | **0.15** | **0.50** | **0.76** | **0.76** | 0.12 |
| | | sd | 0.02 | 0.03 | 0.11 | 0.13 | 0.09 | 0.06 |

T= treatment. C = control. Hydroponic solutions were half-strength Hoagland (pH 5.2) and T1-8 contained 2.25 mg $L^{-1}$ Cd, administered as $CdCl_2$. Where present, the concentrations of Fe and Zn in the hydroponic solutions were 2.38 mg $L^{-1}$ and 25 µg $L^{-1}$, respectively, and were administered as Fe-EDTA and $ZnSO_4$. The foliar treatments were sprays of 0.1% solutions of Fe- and Zn-EDTA. *Insufficient sample for Cd isotope composition analysis.

**Table S5:** Mass of Cd in roots, stems, leaves and total plants, translocation factors and fractions of total Cd in plant organs.

| T | Nutrient Solutions | | Cd (µg) | | | | | | | | TF | | Fraction of total Cd | | | | | |
|---|---|---|---|---|---|---|---|---|---|---|---|---|---|---|---|---|---|---|
| # | Hydroponic | Foliar | Roots | sd | Stems | sd | Leaves | sd | Total | sd | % | sd | Roots | sd | Stems | sd | Leaves | sd |
| 1 | Fe, Zn | - | 38.8 | 2.4 | 51.3 | 8.7 | 52.1 | 3.1 | 142 | 9 | 268 | 47 | 0.27 | 0.04 | 0.36 | 0.04 | 0.37 | 0.00 |
| 2 | Fe, Zn | Fe | 44.6 | 10.1 | 49.5 | 9.5 | 92.8 | 17.9 | 187 | 38 | 324 | 11 | 0.24 | 0.01 | 0.27 | 0.00 | 0.50 | 0.00 |
| 3 | Fe, Zn | Zn | 38.4 | 1.1 | 36.5 | 10.8 | 66.0 | 14.6 | 141 | 24 | 268 | 74 | 0.28 | 0.06 | 0.26 | 0.03 | 0.47 | 0.02 |
| 4 | Zn | - | 44.4 | 0.7 | 20.0 | 6.1 | 17.3 | 7.6 | 82 | 14 | 84 | 30 | 0.55 | 0.09 | 0.24 | 0.03 | 0.21 | 0.06 |
| 5 | Zn | Fe | 51.4 | 4.8 | 29.8 | 1.6 | 19.4 | 2.7 | 101 | 0 | 96 | 17 | 0.51 | 0.05 | 0.30 | 0.02 | 0.19 | 0.03 |
| 6 | Fe | - | 39.3 | 6.2 | 42.9 | 29.5 | 36.2 | 32.2 | 118 | 68 | 191 | 127 | 0.38 | 0.16 | 0.35 | 0.05 | 0.27 | 0.12 |
| 7 | Fe | Zn | 32.9 | 5.4 | 43.8 | 5.9 | 45.7 | 15.6 | 122 | 27 | 270 | 21 | 0.27 | 0.02 | 0.36 | 0.03 | 0.37 | 0.05 |
| 8 | - | - | 31.6 | 5.3 | 13.6 | 5.7 | 13.9 | 15.0 | 59 | 15 | 94 | 81 | 0.57 | 0.24 | 0.22 | 0.04 | 0.21 | 0.20 |
| | | **Mean** | **40** | **5** | **36** | **10** | **43** | **14** | **118** | **22** | **199** | **51** | **0.38** | **0.08** | **0.29** | **0.03** | **0.32** | **0.06** |
| | | sd | 6 | 3 | 14 | 8 | 27 | 10 | 40 | 16 | 96 | 40 | 0.14 | 0.08 | 0.06 | 0.02 | 0.12 | 0.07 |
| | | Min | 32 | 1 | 14 | 2 | 14 | 3 | 59 | 0 | 84 | 11 | 0.24 | 0.01 | 0.22 | 0.00 | 0.19 | 0.00 |
| | | Max | 51 | 10 | 51 | 29 | 93 | 32 | 187 | 49 | 324 | 127 | 0.57 | 0.24 | 0.36 | 0.06 | 0.50 | 0.20 |

T = treatment. Hydroponic solutions were half-strength Hoagland (pH 5.2) and contained 2.25 mg $L^{-1}$ Cd, administered as $CdCl_2$. Where present, the concentrations of Fe and Zn in the hydroponic solutions were 2.38 mg $L^{-1}$ and 25 µg $L^{-1}$ and were administered as Fe-EDTA and $ZnSO_4$. The foliar treatments were sprays of 0.1% solutions of Fe-EDTA and Zn-EDTA. Values are averages of two replicate plants per treatment. TF = translocation factor, which is calculated by dividing the total Cd in the shoots (leaves + stems) by the total Cd in the roots, multiplied by 100.

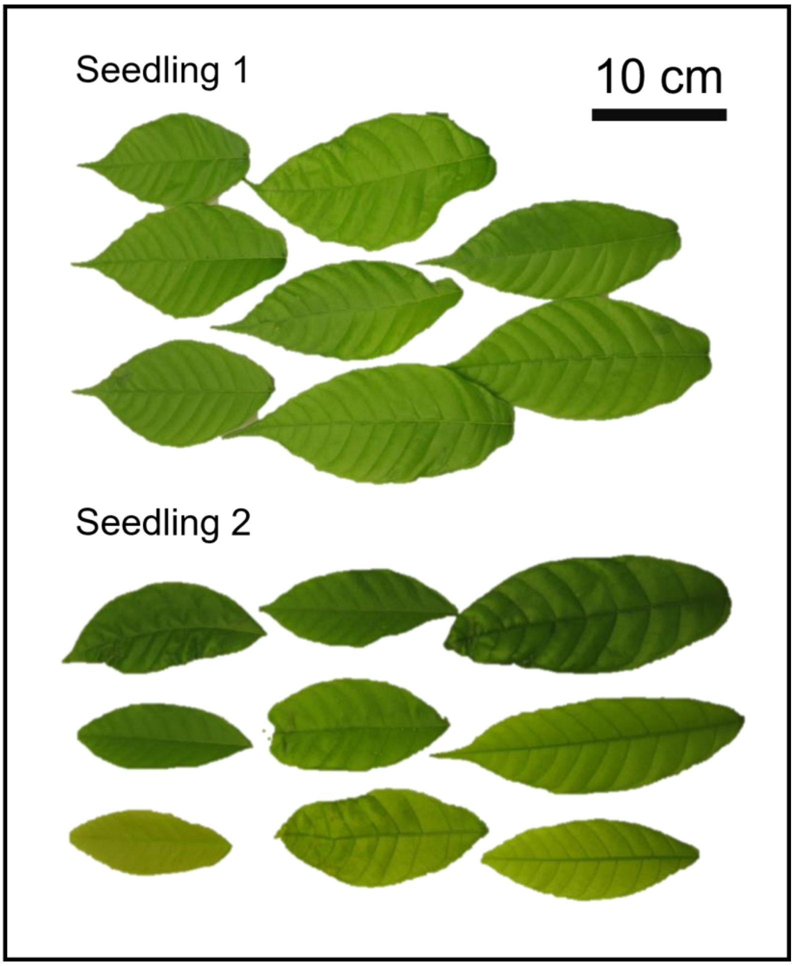


**Figure S1:** Post-harvest photograph of leaves from two cacao seedlings grown in hydroponics with the addition of 20 μM $CdCl_2$, at the Controlled Environment Laboratories (Reading, UK). No necrosis (death of leaf cells) or chlorosis (yellowing of the leaf from lack of chlorophyl) is evident.

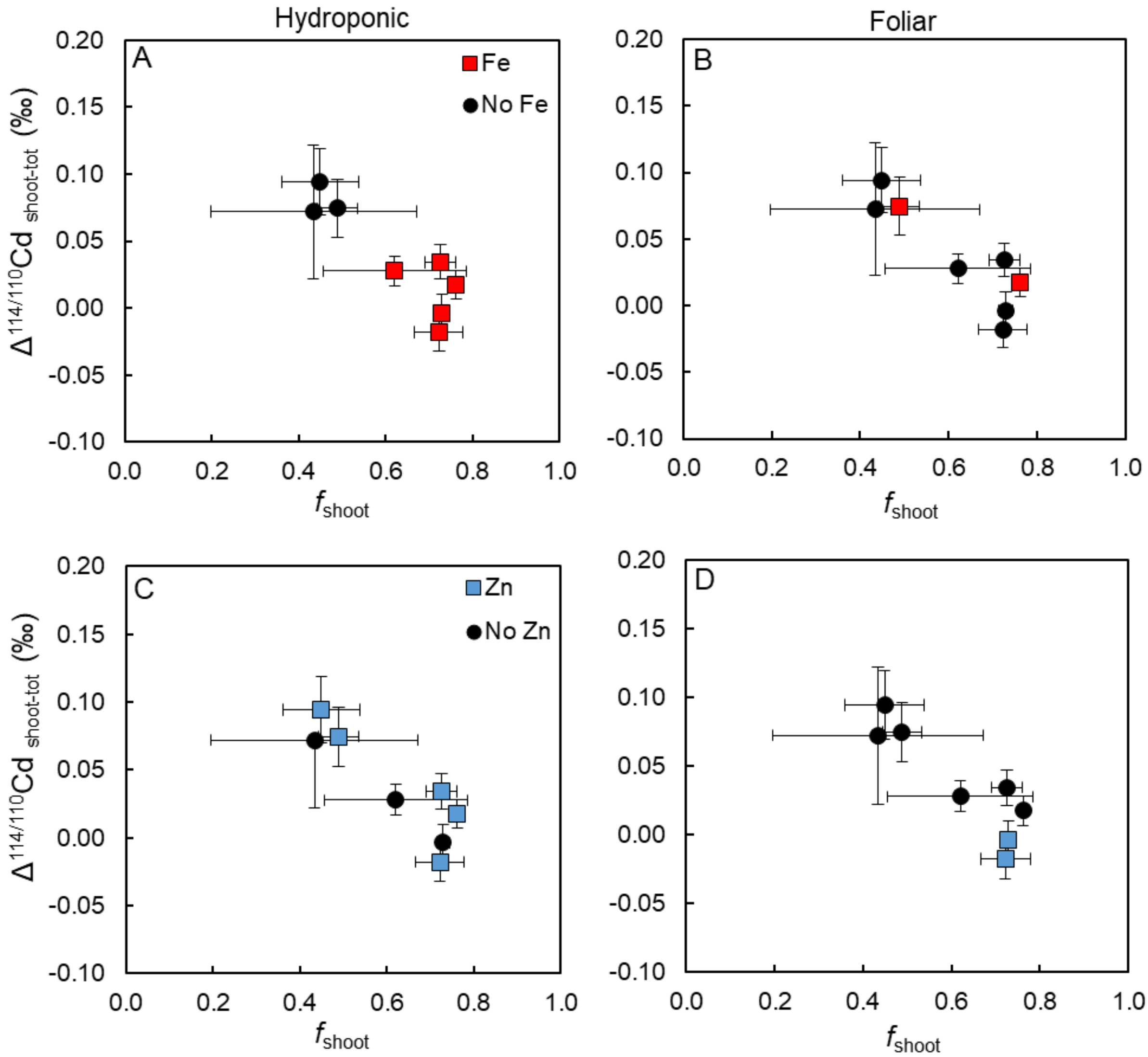


**Figure S2:** Apparent Cd isotope fractionation between shoots and total plant versus fraction (*f*) of total plant Cd in these shoots. Plots A and B highlight hydroponic and foliar Fe treatments, respectively. Plots C and D highlight hydroponic and foliar Zn treatments, respectively. Values are averages of two replicates for each treatment. Average isotope compositions were calculated using inverse-variance weighting.

**References:**

[1] Abouchami, W. et al. A Common Reference Material for Cadmium Isotope Studies - NIST SRM 3108. *Geostand. Geoanalytical Res.* 37, 5–17 (2013.

[2] Moore, R. E. T. et al. Cadmium isotope fractionation reveals genetic variation in Cd uptake and translocation by *Theobroma cacao* and role of natural resistance-associated macrophage protein 5 and heavy metal ATPase-family transporters. *Hortic. Res.* 7, 71 (2020).

[3] Lv, W., Yin, H., Liu, M., Huang, F., Yu, H. Effect of the Dry-Ashing Method on Cadmium Isotope Measurements in Soil and Plant Samples. *Geostand. Geoanalytical Res.* 45, 1–12 (2020).

[4] Borovička, J., Ackerman, L., Rejšek, J., Cadmium isotopic composition of biogenic certified reference materials determined by thermal ionization mass spectrometry with double spike correction. *Talanta*, 221, 121389 (2021).

[5] Wiggenhauser, M. et al. Cadmium transfer in contaminated soil-rice systems: Insights from solid-state speciation analysis and stable isotope fractionation. *Environ. Pollut.* 269, 115934 (2021)
